# Improved sugar co-utilisation by encapsulation of a recombinant *Saccharomyces cerevisiae* strain in alginate-chitosan capsules

**DOI:** 10.1186/1754-6834-7-102

**Published:** 2014-07-03

**Authors:** Johan O Westman, Nicklas Bonander, Mohammad J Taherzadeh, Carl Johan Franzén

**Affiliations:** 1 School of Engineering, University of Borås, 501 90 Borås, Sweden; 2 Chemical and Biological Engineering, Industrial biotechnology, Chalmers University of Technology, 412 96 Göteborg, Sweden

## Abstract

**Background:**

Two major hurdles for successful production of second-generation bioethanol are the presence of inhibitory compounds in lignocellulosic media, and the fact that *Saccharomyces cerevisiae* cannot naturally utilise pentoses. There are recombinant yeast strains that address both of these issues, but co-utilisation of glucose and xylose is still an issue that needs to be resolved. A non-recombinant way to increase yeast tolerance to hydrolysates is by encapsulation of the yeast. This can be explained by concentration gradients occuring in the cell pellet inside the capsule. In the current study, we hypothesised that encapsulation might also lead to improved simultaneous utilisation of hexoses and pentoses because of such sugar concentration gradients.

**Results:**

*In silico* simulations of encapsulated yeast showed that the presence of concentration gradients of inhibitors can explain the improved inhibitor tolerance of encapsulated yeast. Simulations also showed pronounced concentration gradients of sugars, which resulted in simultaneous xylose and glucose consumption and a steady state xylose consumption rate up to 220-fold higher than that found in suspension culture. To validate the results experimentally, a xylose-utilising *S. cerevisiae* strain, CEN.PK XXX, was constructed and encapsulated in semi-permeable alginate-chitosan liquid core gel capsules. In defined media, encapsulation not only increased the tolerance of the yeast to inhibitors, but also promoted simultaneous utilisation of glucose and xylose. Encapsulation of the yeast resulted in consumption of at least 50% more xylose compared with suspended cells over 96-hour fermentations in medium containing both sugars. The higher consumption of xylose led to final ethanol titres that were approximately 15% higher. In an inhibitory dilute acid spruce hydrolysate, freely suspended yeast cells consumed the sugars in a sequential manner after a long lag phase, whereas no lag phase was observed for the encapsulated yeast, and glucose, mannose, galactose and xylose were utilised in parallel from the beginning of the cultivation.

**Conclusions:**

Encapsulation of xylose-fermenting *S. cerevisiae* leads to improved simultaneous and efficient utilisation of several sugars, which are utilised sequentially by suspended cells. The greatest improvement is obtained in inhibitory media. These findings show that encapsulation is a promising option for production of second-generation bioethanol.

## Background

Second-generation bioethanol has long been suggested as a contender to become the main type of renewable liquid fuel [1]. Nevertheless, there are issues with its production that still limit its commercialisation. One of the main problems is the issue of inhibitors produced during the pretreatment and hydrolysis of the raw material into fermentable sugars. Another problem is the fact that pentoses are not fermentable by wild-type *Saccharomyces cerevisiae*[2], although there are a few exceptional cases of slow consumption [3]. The most popular approach to this problem has been to create recombinant strains of *S. cerevisiae* utilising xylose, arabinose or a mixture of the two [4]. However, these strains still have the problem of poor simultaneous co-utilisation of the pentoses together with hexoses [5]. Xylose will not be consumed in considerable amounts until the concentration of glucose is low [6,7]. The reason for this is that there are no specific pentose transporters in *S. cerevisiae*, and pentoses are instead transported by the native hexose transporters [8,9]. However, these transporters have higher affinity for glucose, and therefore there is a strong preference for glucose uptake as long as it is present.

Cell retention through immobilization of the cells can be used as a means to increase the volumetric productivity of biological processes [10]. Numerous examples of immobilization of various microbial cells in alginate beads can be found in the literature, for example, the immobilization of *Debaryomyces hansenii* for xylitol production [11,12] or of *S. cerevisiae* for ethanol production [13]. Immobilization produces several benefits to the process, such as easier cell reuse at high biomass concentration. Cell encapsulation in a semi-permeable membrane differs from bead immobilization in that the cells grow inside a liquid core, forming a dense cell pellet inside the capsule rather than being dispersed in the pores of the alginate matrix [10]. Encapsulation appears to be a good solution to the first of the aforementioned problems, the inhibitor tolerance. It has been shown that encapsulation of the yeast increases its tolerance towards convertible inhibitors such as the furan aldehydes. This effect is believed to be a result of concentration gradients, which are formed when cells close to the membrane convert inhibitors; the cells closer to the core of the pellet are then surrounded by sub-inhibitory levels of the inhibitors, and can still ferment the medium efficiently [14]. It has also been shown on a proteomic level that there are cells in the capsule that are starved despite the presence of high ‘extracapsular’ levels of glucose [15]. This is most likely an effect of concentration gradients of glucose occurring throughout the cell pellet inside the capsules, owing to consumption of glucose by the yeast cells and limitations in mass transfer. Such concentration gradients could hypothetically also promote glucose and xylose co-utilisation. Inside a capsule, some cells will experience a low glucose concentration at the same time that other cells experience a high glucose concentration; however, because glucose inhibits xylose consumption all cells may experience relatively high xylose concentrations. It is plausible to assume that the co-utilisation of the sugars will be improved in such a system, because the cells that experience low glucose levels will take up more xylose.

It has been shown that the most efficient transport of xylose is achieved by strains overexpressing single hexose transporter genes in the order *HXT7* > *HXT5* > *GAL2* > *HXT1* > *HXT4*[9]. *HXT7* was also reported to be the best xylose transporter in another study [8]. Encapsulation of yeast has been shown to lead to higher expression of Hxt6/7p, at glucose levels outside the capsules of more than 10 g/l [15]. This is a strong indication that cells in different parts of the capsule have differences in physiology, in that some cells can sense low while others sense high concentrations of glucose. It also indicates that xylose uptake may be stimulated by encapsulation.

Based on this hypothesis, we simulated the sugar consumption of cells in a capsule half-filled with yeast, using finite element modelling, and a kinetic model for glucose and xylose uptake and conversion of furfural and 5-hydroxymethylfurfural (HMF). The simulations showed that the simultaneous utilisation of glucose and xylose would indeed benefit from encapsulation, especially in an inhibitory medium. To validate these results, a xylose-utilising strain of yeast was constructed and encapsulated. The fermentation performance was compared with that of the same yeast in suspended culture in defined inhibitory media and dilute acid spruce hydrolysate.

## Results and discussion

### Finite element modelling shows increased co-consumption due to encapsulation

**Figure 1:**
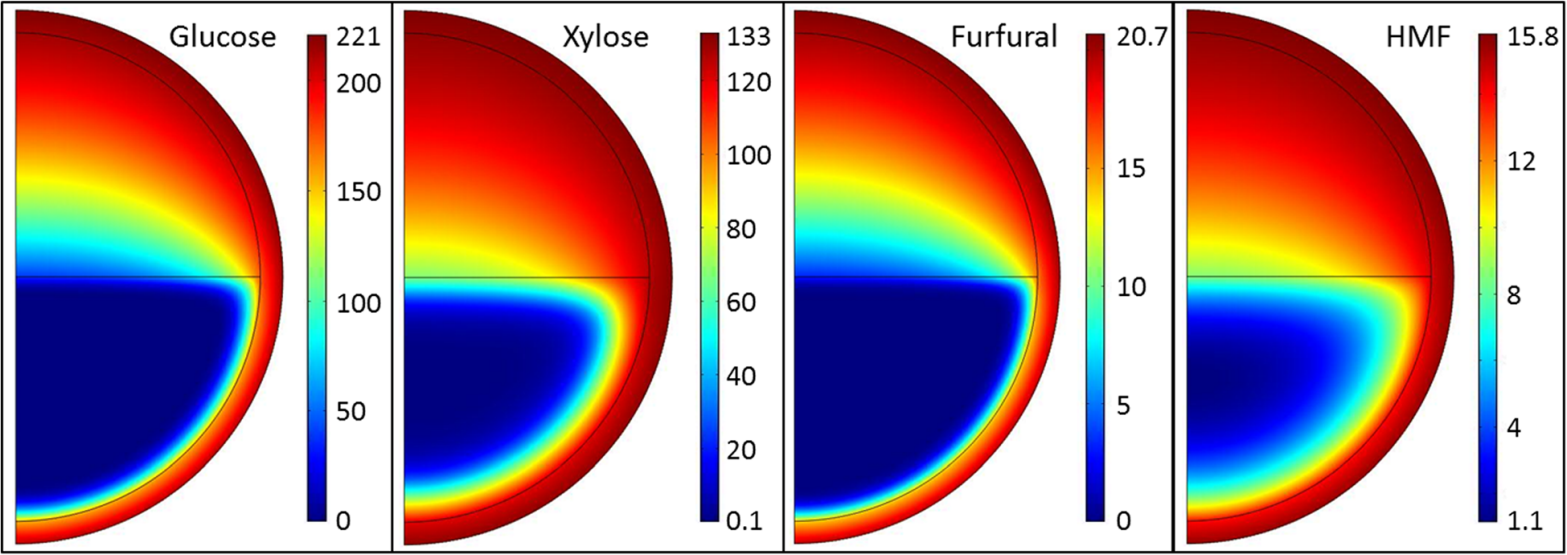
**Simulated concentration gradients in capsules.** Simulated concurrent local concentrations of glucose, xylose, furfural and 2-hydroxymethylfurfural (HMF) at steady state in liquid core capsules half-filled with cells, calculated using finite element modelling in 2D axisymmetrical geometry and base case parameter values. Reactions occurred only in the cell pellet in the lower half of the capsule. Numbers are in mmol/l. The maximum values were equal to the concentrations in the surrounding medium. For details, see Materials and Methods.

Mathematical modelling was implemented to visualise the effect of mass transport resistances on the concentration profiles of glucose, xylose, HMF and furfural in a capsule, and to analyse the sensitivity of the sugar consumption rates to different characteristics of the system.The base case model of the diffusion and reaction rates resulted in very low concentrations of glucose and furfural in large parts of the cell pellet (Figure 1). In the centre of the pellet, the steady state glucose and furfural concentrations were 0 mM because of rapid conversion of these compounds by the cells closer to the surface of the cell pellet. By contrast, the minimum concentrations of xylose and HMF were 0.1 mM and 1.1 mM, respectively, because of the lower conversion rates. These results indicate that the overall conversion rates of glucose and furfural are diffusion-limited, whereas the conversion rates of xylose and HMF may be limited by the intrinsic reaction rate. They also indicate that there are zones in the cell pellet where the inhibition of xylose consumption by glucose, furfural and HMF should be low.

Efficiency factors were calculated to compare the simulated results for encapsulated cells with well-mixed conditions and suspended cells. The efficiency factors were obtained by dividing the overall conversion rates with the conversion rates obtained using 10^5^ times higher effective diffusivities and otherwise identical parameter values. We verified that the higher diffusivities abolished all concentration gradients in the capsule and cell pellet and, therefore, these correspond to well-mixed conditions, with all concentrations being constant and equal to the external bulk concentrations. The calculated efficiency factors showed that the encapsulation increased the xylose consumption by a factor of 39.8, and reduced the glucose consumption by a factor of 0.33, compared with well-mixed conditions under the base case conditions (Table 1). The efficiency factor for xylose was much larger than 1 in all the tested cases, indicating that xylose consumption always benefits from encapsulation. The straightforward explanation for this is that encapsulation or more precisely, the tight agglomeration of cells, reduces local glucose concentration, which leads to lower inhibition of the xylose uptake.

**Table 1:** **Efficiency factors (*β***_***j***_**), rate response coefficients (**$C_{P}^{R_{j}}$**) and efficiency response coefficients (**$C_{P}^{\beta_{j}}$**) for glucose and xylose for the base case and various simulated system changes**

**Condition**	**Modified parameters ( *P *)**	***β***_***glucose***_	***β***_***xylose***_	$C_{P}^{R_{\mathrm{glucose}}}$	$C_{P}^{R_{\mathrm{xylose}}}$	$C_{P}^{\beta_{\mathrm{glucose}}}$	$C_{P}^{\beta_{\mathrm{xylose}}}$
Base case	See Table 3 and Figure 1	0.33	39.8	–	–	–	–
High xylose	*c*_*x*_ = 40 g/l	0.35	36.6	–	–	–	–
High furfural and HMF	*c*_*f*_ = 4 g/l; *c*_*h*_ = 4 g/l	0.46	56.8	–	–	–	–
No furfural and HMF	*c*_*f*_ = 0 g/l; *c*_*h*_ = 0 g/l	0.20	23.5	–	–	–	–
Strong inhibition by furfural and HMF	*K*_*if*_ =5 mM; *K*_*ih*_ = 10 mM	1.69	222	–	–	–	–
Biomass concentration	*C*_*cells*_ = 270 g/l	0.35	41.7	0.38	0.55	−0.62	−0.45
	*C*_*cells*_ = 330 g/l	0.31	38.1				
Maximum glucose uptake rates	*v*_*gmax*, *L*_ = 5.4 mmol/g/h; *v*_*gmax*, *H*_ = 1.8 mmol/g/h	0.35	38.5	0.33	0.31	−0.67	0.31
	*v*_*gmax*, *L*_ = 6.6 mmol/g/h; *v*_*gmax*, *H*_ = 2.2 mmol/g/h	0.31	41.0				
Maximum xylose uptake rate	*v*_*xmax*_ = 5.4 mmol/g/h	0.33	43.3	0.01	0.20	0.01	−0.80
	*v*_*xmax*_ = 6.6 mmol/g/h	0.33	36.9				
Inhibition of glucose uptake by xylose	*K*_*ix*_ = 32.4 mM	0.33	39.5	0.07	0.08	−0.09	0.08
	*K*_*ix*_ = 39.6 mM	0.32	40.1				
Inhibition of glucose and xylose uptake by furfural and HMF	*K*_*if*_ =36 mM; *K*_*ih*_ = 72 mM	0.34	41.8	0.09	0.06	−0.42	−0.45
	*K*_*if*_ =44 mM; *K*_*ih*_ =88 mM	0.32	38.2				
Inhibition of xylose uptake by glucose	*K*_*ig*_ =0.36 mM	0.33	43.9	0.00	0.07	0.00	−0.93
	*K*_*ig*_ =0.44 mM	0.33	36.5				
Effective diffusivity in the cell pellet	*D*_*c,glucose*_ = 1.52 × 10^-10^ m^2^/s; *D*_*c,xylose*_ = 1.73 × 10^-10^ m^2^/s; *D*_*c,furfura*l_ = 2.52 × 10^-10^ m^2^/s; *D*_*c,HMF*_ = 2.39 × 10^-10^ m^2^/s	0.32	38.2	0.34	0.39	0.34	0.39
	*D*_*c,glucose*_ = 1.86 × 10^-10^ m^2^/s; *D*_*c,xylose*_ = 2.11 × 10^-10^ m^2^/s; *D*_*c,furfura*l_ = 3.08 × 10^-10^ m^2^/s; *D*_*c,HMF*_ = 2.92 × 10^-10^ m^2^/s	0.34	41.3				

HMF, 5-hydroxymethylfurfural.

By contrast, the efficiency factors for glucose uptake were generally less than 1, indicating that mass transport becomes limiting for the glucose consumption (Table 1). However, when we increased the inhibition of glucose metabolism by furfural and HMF by decreasing the inhibition constants, glucose consumption also benefitted from the encapsulation. When the inhibition constants were set to *K*_*if*_ = 5 mM and *K*_*ih*_ = 10 mM, encapsulation increased the glucose and xylose consumption rates by 1.69 and 222 times, respectively, compared with well-mixed conditions.

Many of the parameter values used were rather uncertain. For the effective diffusivities, conservative estimates of the mass transfer resistances were used. For the cell pellet, the effective diffusivities were assumed to be 25% of the diffusivities in the surrounding liquid. In comparison, the effective diffusivity of glucose in flocs (that is, unencapsulated cell pellets) of *S. cerevisiae* NRRL Y265 has been shown to be 7 to 17% of that in the surrounding water [16]. Furthermore, the external mass transfer resistance was ignored, and the diffusivities in the gel membrane and liquid core were assumed to be close to or equal to the diffusivity in water [17-20]. All these assumptions should lead to underestimated concentration gradients (that is, overestimated concentrations), inside the capsules, and therefore, the predicted effects of encapsulation should be conservative estimates. Furthermore, it would be very difficult to identify and validate, for example, *K*_*M*_ values and maximum glucose uptake rates, as these inevitably vary along the radius of the cell pellet, owing to the varying glucose concentration and the consequential differential expression of the hexose transporters.

Rate response coefficients and efficiency response coefficients were calculated in order to investigate the sensitivity of the simulation results to changes in different parameters (Table 1). The glucose consumption rate was clearly increased at higher diffusivities, with rate response coefficient $C_{D_{c}}^{R_{\mathrm{Glucose}}}\;=\;0.34$. It was also clearly increased by higher biomass concentration in the pellet and by higher maximum glucose uptake rates ($C_{C_{\mathrm{cells}}}^{R_{\mathrm{Glucose}}}\;=\;0.38$, $C_{v_{\mathrm{gmax}}}^{R_{\mathrm{Glucose}}}\;=\;0.33$). However, although increased diffusivity also led to an increased efficiency factor for glucose ($C_{D_{c}}^{\beta_{\mathrm{Glucose}}}\;=\;0.34$), increased biomass concentration and maximum rates actually decreased the efficiency factor ($C_{C_{\mathrm{cells}}}^{\beta_{\mathrm{Glucose}}}\;=-0.62$, $C_{v_{\mathrm{gmax}}}^{\beta_{\mathrm{Glucose}}}\;=-0.67$). This clearly shows that the glucose consumption rate is diffusion-limited. The glucose uptake efficiency factor increased in response to inhibition by furfural and HMF, and to a smaller extent in response to increased xylose inhibition. At sufficiently strong inhibition, even glucose uptake benefits from encapsulation, as already mentioned above.

The xylose consumption rate was even more sensitive to increased biomass concentration and equally sensitive to *v*_*gmax*_, but for xylose the efficiency response coefficient increased in response to *v*_*gmax*_. Moreover, the efficiency factor for the xylose consumption increased with stronger inhibition (that is, lower *K*_*i*_) by glucose, furfural and HMF ($C_{K_{\mathrm{ig}}}^{\beta_{\mathrm{Xylose}}}\;=-0.93$, $C_{K_{\mathrm{if}},K_{\mathrm{ih}}\;}^{\beta_{\mathrm{Xylose}}}\;=-0.45$). In summary, encapsulation becomes very favourable for xylose consumption if the inhibition effects are strong and the glucose concentration in the capsule can be kept low.

### Construction of the xylose-fermenting *S. cerevisiae* CEN.PK XXX

A xylose-fermenting strain, which we named CEN.PK XXX, was constructed by overexpression of the native *RPE1*, *TAL1*, *RKI1* and *XKS1* genes, and insertion of codon-optimised *XYL1* and *XYL2* genes from *Scheffersomyces stipitis* (formerly *Pichia stipitis*) into the genome of *S. cerevisiae* CEN.PK 122 MDS. The cells were shown to assimilate xylose in repeated rounds of dilutions to OD_600_ = 0.1 and semi-aerobic growth up to OD_600_ = 12 to 13 in 3 days.

### Encapsulation of a xylose-fermenting *S. cerevisiae* to validate simulation results

To test the hypothetical results of the simulations, *S. cerevisiae* CEN.PK XXX was encapsulated and utilised for anaerobic batch cultivations in various media. The fermentations were operated for 96 hours, except for those with suspended yeast in medium without xylose, which were stopped after 30 hours, because all the glucose was consumed by that time.

**Figure 2:**
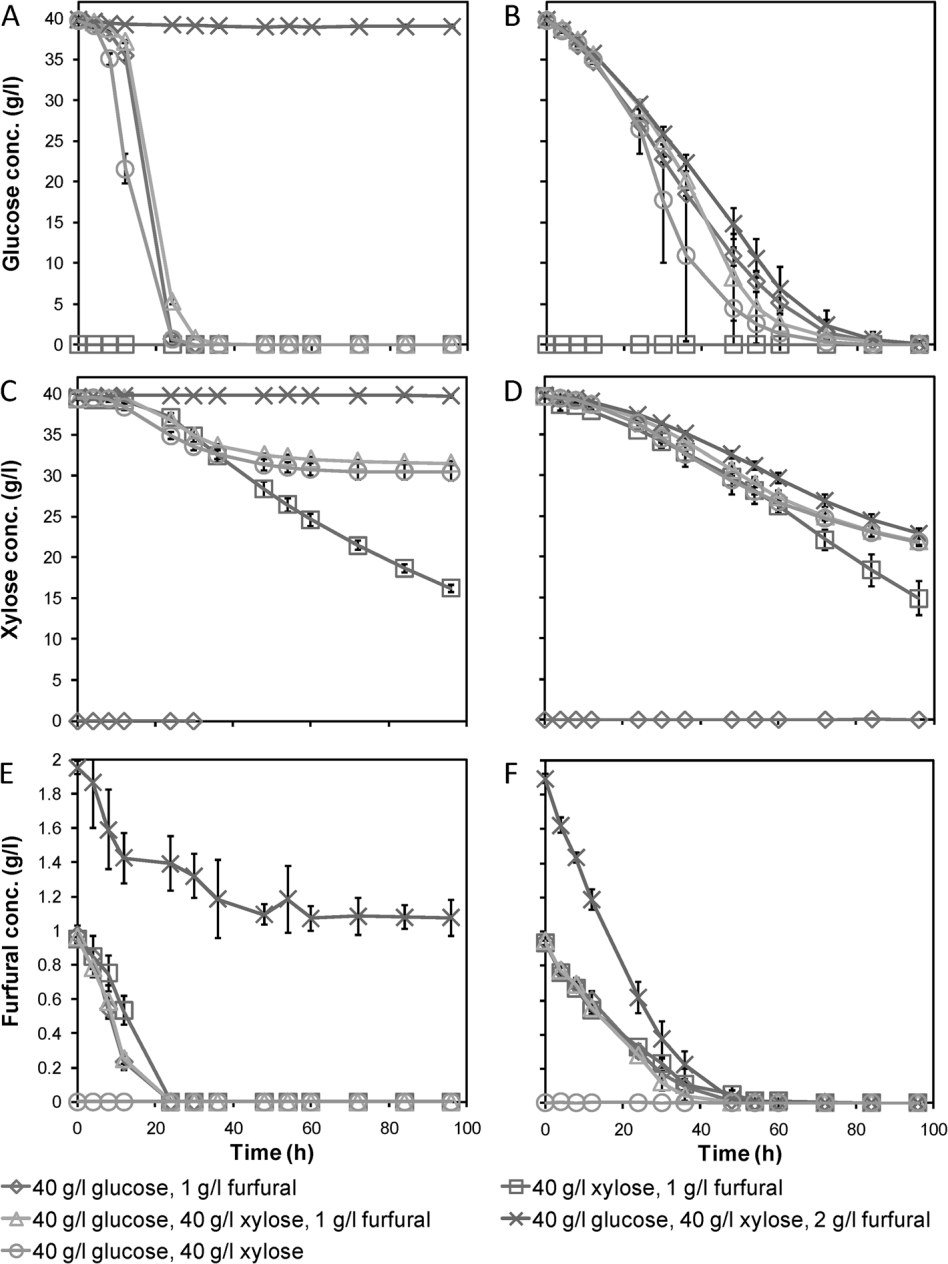
**Concentrations of glucose, xylose and furfural during fermentation of defined media by suspended and encapsulated xylose-fermenting yeast. (A–F)** Concentrations of glucose, xylose and furfural for **(A, C, E)** freely suspended and **(B, D, F)** encapsulated *Saccharomyces cerevisiae* CEN.PK XXX in anaerobic batch cultures on defined media containing different initial concentrations of glucose, xylose and furfural. Average values ± 1 standard deviation, n = 3.

The encapsulated cells did not show a lag phase prior to glucose consumption, whereas a lag phase was observed for the freely suspended cells in all cases except when furfural was absent (Figure 2A,B). This is in agreement with previous observations [21]. The absence of a lag phase for encapsulated cells would be expected if there were concentration gradients of inhibitors inside the capsules [14]. The furfural would only inhibit the cells close to the membrane for encapsulated cells. Sugars that diffuse through the capsule membrane and the cell pellet would be fermented by cells further into the pellet already at the beginning of the cultivation.For the suspended cells, rapid glucose consumption began only after the furfural was present at concentrations below 0.7 to 0.8 g/l, which occurred 8 to 24 hours into the cultivations (Figure 2A,C,E). Furthermore, suspended CEN.PK XXX at this cell concentration did not tolerate a furfural concentration of 2 g/l. The cells managed to convert less than half of the furfural present, without visible glucose or xylose consumption. However, when encapsulated, the cells in the medium with 2 g/l furfural exhibited only a somewhat slower uptake of both glucose and xylose compared with cells in the other media (Figure 2B,D).

### Improved simultaneous co-utilisation of glucose and xylose by encapsulation

The xylose consumption was rather similar between the encapsulated and freely suspended yeast cells when xylose was the only carbon source (Figure 2C,D). Hence, the diffusion limitations observed for glucose, which led to slower utilisation, was not a limitation for xylose utilisation, confirming the results of the simulations.

**Figure 3:**
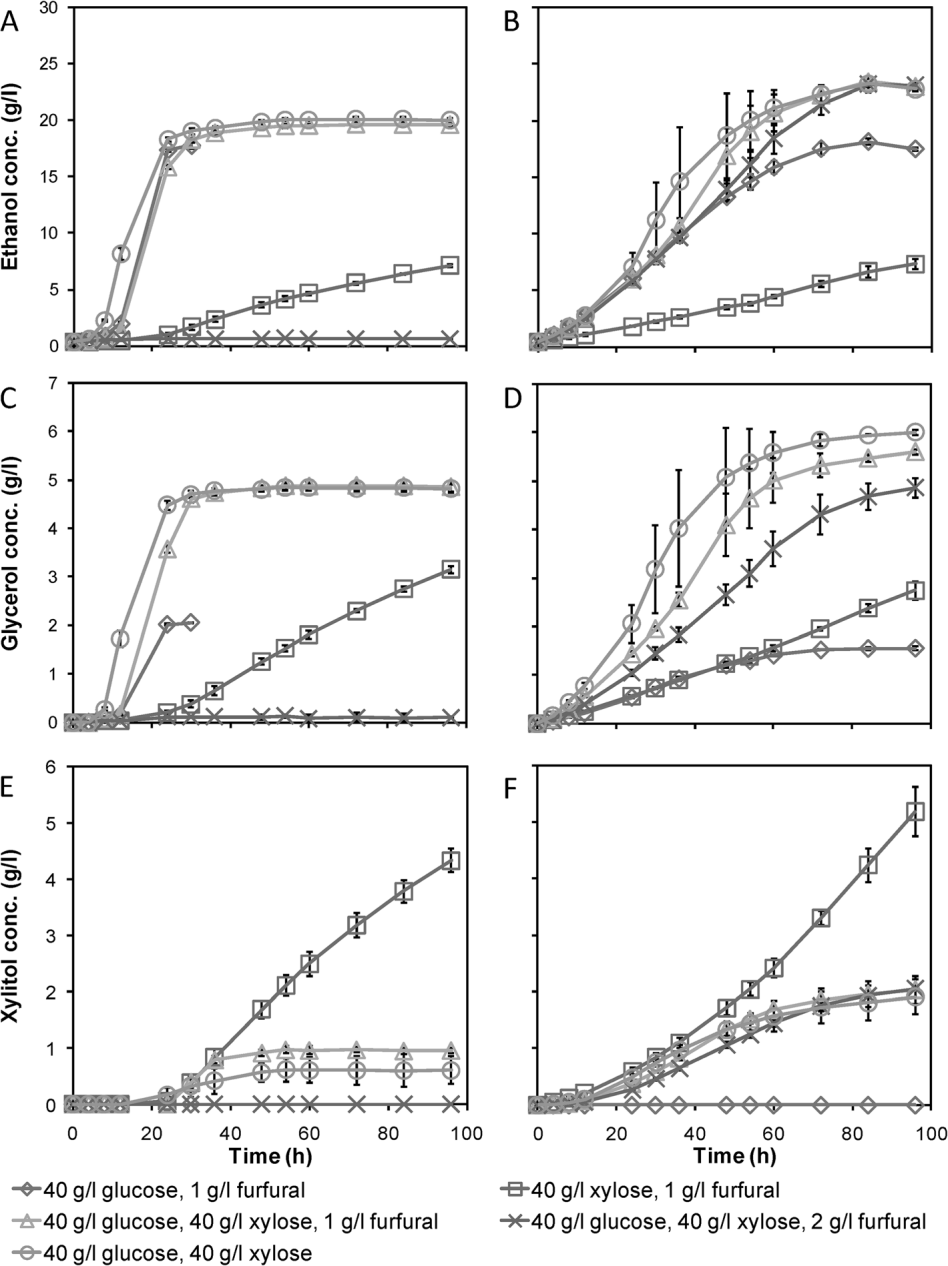
**Concentrations of ethanol, glycerol and xylitol during fermentation of defined media by suspended and encapsulated xylose-fermenting yeast. (A–F)** Concentrations of ethanol, glycerol and xylitol for **(A, C, E)** freely suspended and **(B, D, F)** encapsulated *Saccharomyces cerevisiae* CEN.PK XXX in anaerobic batch cultures on defined media containing different initial concentrations of glucose, xylose and furfural. Average values ± 1 standard deviation, n = 3.

In media with both xylose and glucose, there were significant differences between the free and encapsulated cells. The suspended cells displayed some co-consumption of the two sugars, with approximately 6.5 g/l reduction in the xylose concentration concomitant with the consumption of all glucose. This consumption was accompanied by a high production of glycerol, but very low accumulation of xylitol (Figure 3). The glycerol production helped to reoxidise NADH, therefore, virtually all xylitol could be converted to xylulose. When the glucose was depleted, xylitol started to accumulate, most likely due to a lack of NAD^+^, as the high glycerol production could not be maintained. Xylose consumption almost ceased at a residual xylose concentration of about 30 g/l, approximately 24 hours after glucose depletion. This slow xylose utilisation after glucose depletion has been observed previously [7]. The exact reason is not known, but tentatively, ceased glucose metabolism may lead to a lack of intermediary metabolites for the initial steps of xylose metabolism and the pentose phosphate pathway, leading to reduced rates [7]. When no more glucose is consumed, there might be a shortage of both NADPH and NAD^+^, generated through the oxidative pentose phosphate pathway and from glycerol production, respectively. Severe redox imbalance problems could then be the reason for the extremely slow xylose consumption after glucose exhaustion, as suggested by Kim *et al*. [22]. Alternatively, simultaneous consumption of glucose and xylose can facilitate more efficient conversion of xylose, xylitol and xylulose into glycolytic intermediates and subsequently into ethanol. Tentatively, the cells are in a highly metabolically active state at the point of glucose depletion, and they might not be able to adapt quickly enough to the rapid drop in NADPH and NAD^+^ expected during pure anaerobic xylose utilisation. Moreover, ATP depletion can occur, if xylose, which at that time was the only carbon source, is used for glycerol production to reoxidise NADH rather than for substrate level phosphorylation in glycolysis. If xylulokinase activity is too high, this can also lead to depletion of ATP [23]. With xylose present as the only carbon and energy source already from the start of the fermentation, the cells probably adapted their metabolism and maintained the redox balance. Therefore, a decrease in xylose consumption rate was not observed in this case (Figure 2C).

The encapsulated CEN.PK XXX fermented xylose from the beginning of the batches, regardless of the initial furfural concentration. A slight reduction in the uptake rate of xylose was observed as the glucose was depleted in the media with mixed carbohydrates (Figure 2D). However, the final uptake of xylose after 96 hours was significantly better for the encapsulated cells than for the freely suspended cells in media containing glucose. The final residual xylose concentrations were 21.4 to 23.4 g/l. Hence, encapsulated *S. cerevisiae* CEN.PK XXX consumed at least 50% more xylose than the freely suspended cells, under the same conditions. This significant difference was also clearly visible in the ethanol production, and resulted in approximately 15% higher final concentrations (Figure 3).

**Figure 4:**
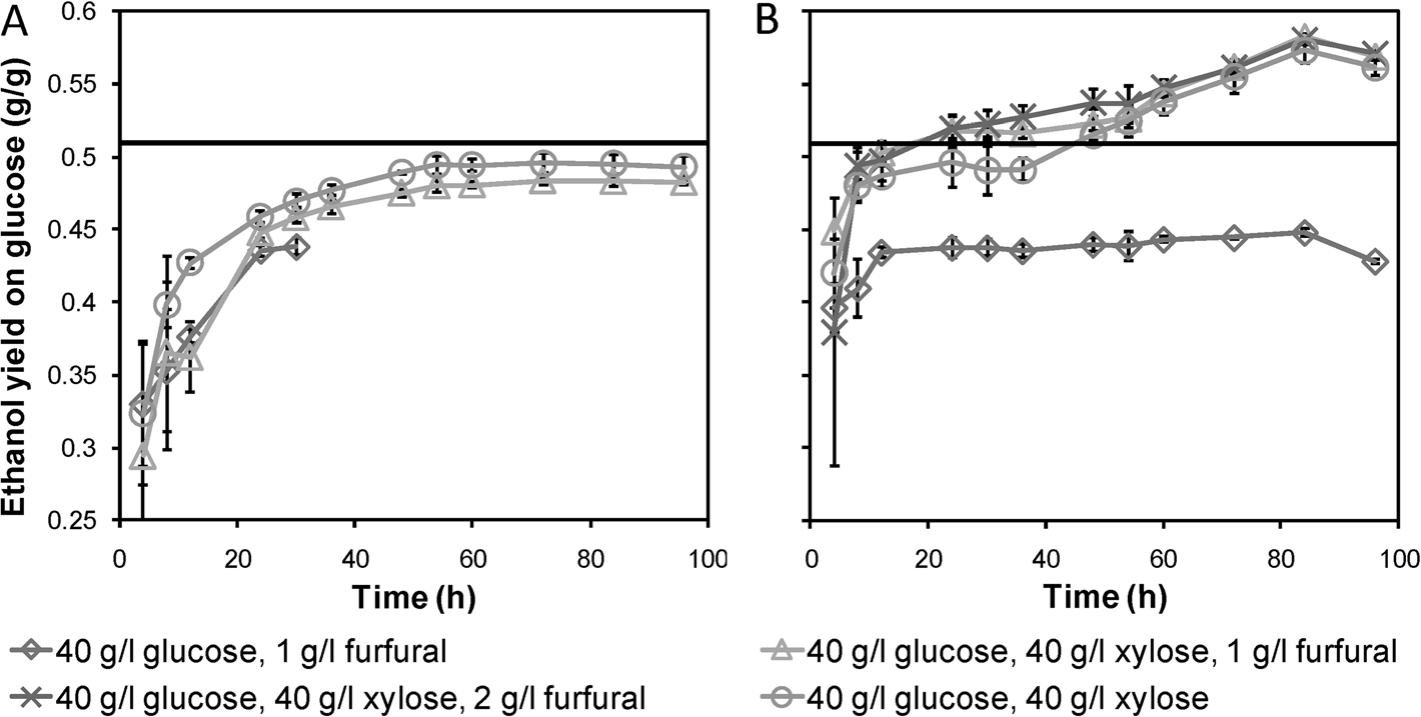
**Cumulative total yield of ethanol on glucose during fermentation of defined media. (A)** Suspended and **(B)** encapsulated xylose-fermenting yeast. The total ethanol yield was calculated from the glucose consumption and total ethanol production from both xylose and glucose from the beginning of the batch to each sample point. The theoretically highest possible ethanol yield on glucose only (0.51 g/g) is indicated by the black line. Yields higher than this level indicate ethanol production from xylose. Average values ± 1 standard deviation, n = 3.

Tentatively, a large portion of the encapsulated cells will have experienced glucose concentrations of zero or close to zero throughout the batches containing glucose and xylose, as observed in the simulations. In this sense, these cells resembled cells in medium with only xylose present, with a balanced metabolism occurring throughout the batch. In contrast to suspended cells, these cells will thus not experience a huge difference upon glucose depletion. Hence, sudden large redox imbalances, or ATP depletion, at the end of the glucose consumption phase are probably not present for most of the encapsulated cells. However, the cells close to the membrane might have been disturbed by the change in metabolism, and this may have caused the small decrease in xylose consumption observed after glucose depletion.The total ethanol yields on consumed glucose during the course of the fermentation show that the encapsulated cells utilised glucose and xylose more simultaneously and more efficiently than the freely suspended yeast, corroborating the results from the simulations (Figure 4). For the suspended yeast, the total ethanol yield on glucose at 24 hours was slightly higher in the mixed carbohydrate cultures than in those with glucose as the sole carbon source. This indicates that co-utilisation had taken place. However, for the encapsulated yeast, the cultures with mixed carbohydrates exhibited a higher ethanol yield throughout the course of the fermentation. Early in the batches, this yield exceeded the theoretical maximum for ethanol production from glucose only (0.51 g/g), as indicated by the black horizontal line in Figure 4. The final ethanol yield on glucose was up to 33% higher with the mixed sugars than with glucose only, clearly showing that the encapsulated yeast produced ethanol from xylose throughout the batches.For the suspended cells, most of the xylose consumption took place at low glucose levels. This was especially the case in the medium without furfural. After 12 hours, almost half of the glucose had been consumed, with only about 1 g/l of xylose consumed simultaneously (Figure 2A,C). By contrast, the encapsulated yeast already showed co-consumption of the two sugars at the high glucose concentrations at the beginning of the cultivations. Furthermore, we observed that the co-utilisation of xylose by the encapsulated cells increased during the course of the fermentations. We suggest this occurs because the capsules fill up with cells, increasing the effect of concentration gradients in the cell pellets.The cells in the cultivation without furfural displayed less co-consumption of the glucose and xylose (Figure 4). Owing to occasional breakage of capsules during the precultivations, yeast cells clinging to the outside of the capsules were sometimes transferred with the capsules into the anaerobic batch cultivations. In some cases, cells grew outside the capsules, especially when no furfural remained in the medium. This led to faster glucose consumption and hence less co-utilisation of xylose. Therefore, the error bars are disproportionately high for zero and low initial concentrations of furfural for the encapsulated yeast. However, this does not compromise the conclusions drawn from this study.

### Product yields

The encapsulated yeast utilised the sugars in a rather similar fashion to the freely suspended yeast (Table 2). However, with only xylose present, the glycerol yield was higher for the encapsulated than the suspended yeast, opposite to the results observed for glucose only both in this study (Table 2) and a previous study [15]. Furthermore, the glycerol yields decreased with increasing furfural concentration for the encapsulated cells. This happens because the furfural acts as a redox sink, reoxidising NADH during its conversion into furfuryl alcohol [24]. This effect was not observed for the suspended cells, because the conversion of furfural occurred before consumption of sugars in those cultivations. The xylitol yields were generally higher for the encapsulated than for the suspended cells (Table 2). This could be an effect of stricter anaerobic conditions for the encapsulated cells, leading to accumulation of more xylitol [5]. Oxygen was not actively stripped from the shake flasks at the start of the batch fermentations, and oxygen would not reach the encapsulated cells as readily as it would reach the freely suspended cells. The higher glycerol yield on xylose, which led to reoxidation of NADH to NAD^+^, which is needed for xylitol oxidation, can be explained in the same way. The ethanol yields on the two sugars were rather similar overall between the two modes of cultivation, with a slightly lower yield for the encapsulated cells as they consumed more xylose. We observed from the results that CEN.PK XXX was able to grow anaerobically on xylose as the sole carbon source at relatively high biomass yields (Table 2). However, it should again be stressed that there was some oxygen present in the flasks at the start of the anaerobic fermentations and the biomass was measured only at the end. The biomass yields were generally lower for the encapsulated cells.

**Table 2:** **Final yields and carbon recoveries in anaerobic batch fermentations of defined media**^**a**^

	**Medium**^**b**^	**Y**_**SGly**_	**Y**_**SAce**_	**Y**_**SXylitol**_	**Y**_**SBiomass**_	**Y**_**SEtOH**_	**Carbon balance (%)**
Freely suspended CEN.PK XXX	G40 F1	52 ± 1	3 ± 0	NA	48 ± 0	438 ± 6	97 ± 1
	X40 F1	83 ± 6	8 ± 3	187 ± 6	41 ± 1	290 ± 0	94 ± 1
	G40 X40	99 ± 1	5 ± 1	67 ± 21	38 ± 0	402 ± 1	94 ± 1
	G40 X40 F1	102 ± 2	3 ± 0	121 ± 9	37 ± 1	402 ± 1	95 ± 1
	G40 X40 F2	NA	NA	NA	NA	NA	NA
Encapsulated CEN.PK XXX	G40 F1	39 ± 1	2 ± 1	NA	47 ± 3	428 ± 2	93 ± 1
	X40 F1	111 ± 0	3 ± 1	209 ± 4	55 ± 2	277 ± 3	92 ± 1
	G40 X40	104 ± 1	3 ± 0	106 ± 15	32 ± 3	387 ± 1	93 ± 2
	G40 X40 F1	97 ± 2	2 ± 0	114 ± 9	30 ± 1	393 ± 1	94 ± 1
	G40 X40 F2	86 ± 2	1 ± 0	122 ± 5	29 ± 2	401 ± 2	94 ± 1

*Abbreviations*: *NA* not applicable.

^a^Yields are shown as mg product per g consumed sugar (average values ± 1 standard deviation, n = 3). Acetate (Y_SAce_), ethanol (Y_SEtOH_), glycerol (Y_SGly_) and biomass (Y_SBiomass_) yields were calculated on total consumed sugars, whereas the xylitol (Y_SXylitol_) yield was calculated on consumed xylose only.

^b^G40: glucose 40 g/l; X40: xylose 40 g/l; F1: furfural 1 g/l; F2: furfural 2 g/l.

### Encapsulation solves two issues of lignocellulosic hydrolysate fermentation

**Figure 5:**
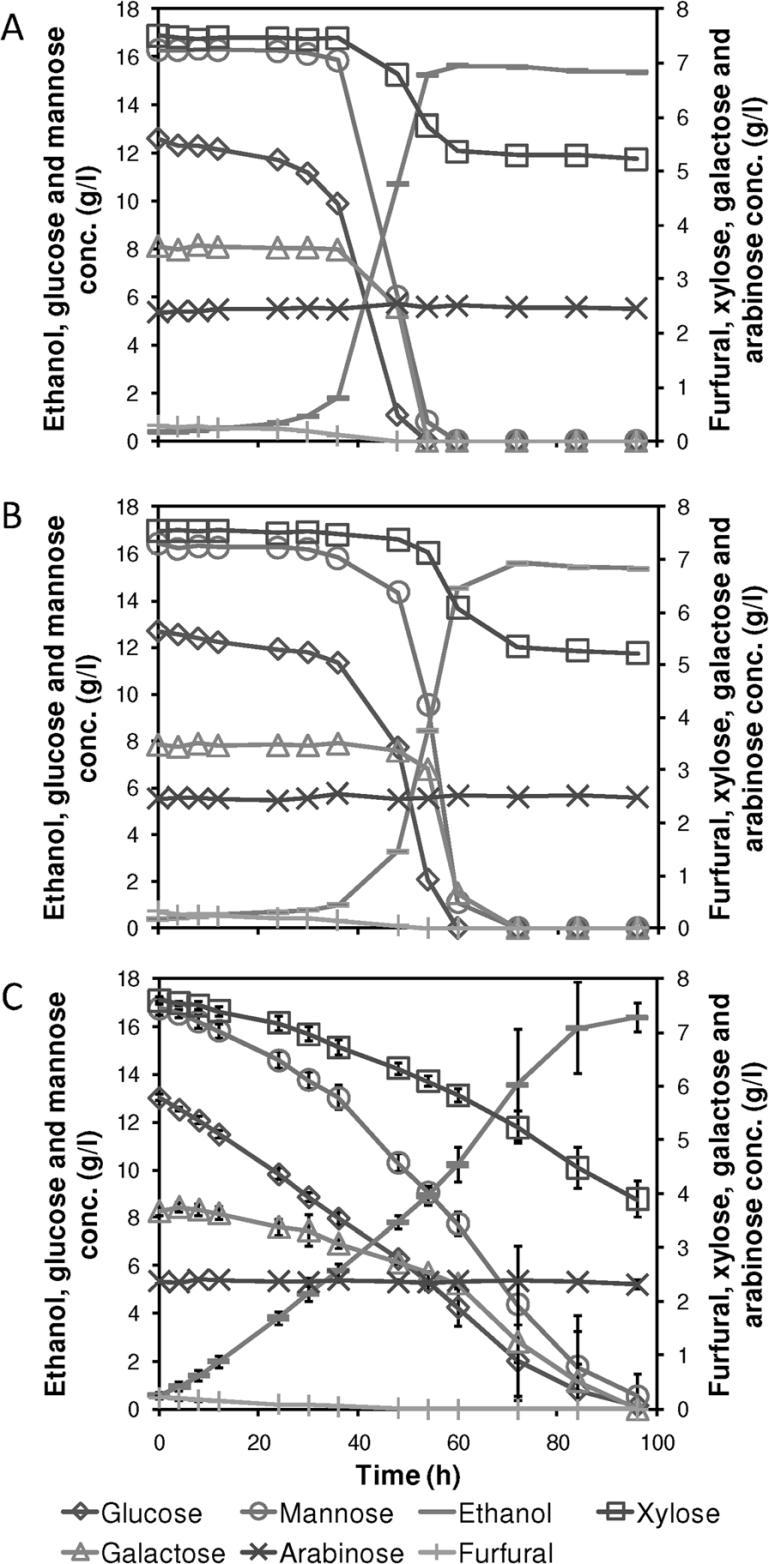
**Concentrations of substrates and products during fermentation of dilute acid spruce hydrolysate. (A, B)** Results for freely suspended *S. cerevisiae* CEN.PK XXX are shown in two separate graphs because of differences in the length of the lag phase. **(C)** Concentrations for encapsulated yeast are shown as average values of triplicate fermentations ± 1 standard deviation.

To investigate the overall performance of encapsulated cells, and as a proof of concept, fermentation of a spruce hydrolysate was investigated with encapsulated and freely suspended *S. cerevisiae* CEN.PK XXX. We observed that the encapsulated cells were able to consume all sugars, except arabinose, starting from the beginning of the fermentation (Figure 5C). This occurred concurrently with *in situ* detoxification by conversion of furfural, and resulted in approximately 7% higher final ethanol concentration for the encapsulated than for the suspended yeast, 16.4 ± 0.6 (n = 3) and 15.4 ± 0.0 (n = 2), respectively (*P* < 0.05, one-tailed *t*-test) (Figure 5).Co-consumption of glucose, mannose, xylose and galactose was not observed for suspended yeast at glucose concentrations above 10 g/l (Figure 5A,B). The suspended yeast did not start to consume notable amounts of any sugar until the hydrolysate was detoxified by conversion of the present furfural and HMF. After full conversion of the furfural, consumption of glucose and mannose occurred rapidly. Consumption of xylose and galactose by the suspended yeast was only observed once the concentration of glucose was low enough. The total amount of xylose consumed was also slightly lower for the freely suspended cells. Xylose consumption stopped shortly after glucose depletion, similar to the observations for the fermentations of defined media.

As a result of encapsulating the relatively sensitive laboratory strain *S. cerevisiae* CEN.PK XXX, the inhibitor tolerance of the system as a whole and its ability to simultaneously co-utilise glucose and xylose were improved. Another benefit of cell encapsulation, which has not been stressed in this work, is that the cells are easily retained in the reactor. Thus, the volumetric productivity can be easily improved by increasing the amount of capsules in the reactor.

## Conclusions

Results from *in silico* simulations and *in vivo* experiments showed that encapsulation of a xylose-fermenting yeast strain increased the simultaneous utilisation of glucose and xylose and improved the tolerance to furfural. All the data indicate that the reason behind these results is that diffusion limitations cause concentration gradients of convertible inhibitors and glucose within the cell pellet. High local cell density, here in the form of encapsulated yeast, can thus help in overcoming two of the remaining obstacles for successful commercialisation of second-generation bioethanol production, namely, fermentation inhibitors and sequential hexose and pentose fermentation.

## Materials and methods

### Mathematical modelling

Diffusion and consumption of glucose, xylose, furfural and HMF by encapsulated cells was simulated using finite element modelling (FEM) and simplified kinetics. The Chemical Reaction Engineering Module in Comsol Multiphysics 4.3 (Comsol AB, Stockholm, Sweden) was used for all simulations.

### Physical description of capsules and meshing for FEM

**Figure 6:**
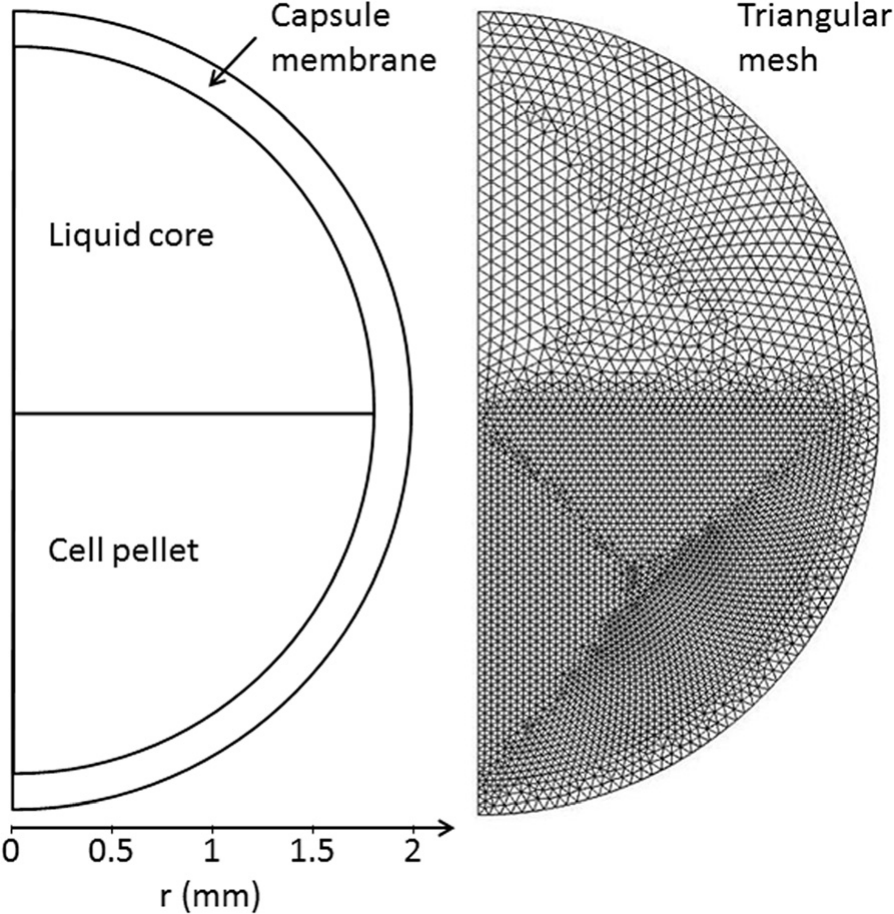
**Geometry and meshing of a capsule for finite element modelling.** Two-dimensional view of an alginate-chitosan capsule half-filled with yeast cells, showing the three computational compartments: the capsule membrane, the liquid core and the cell pellet (left), which were meshed into 781, 1,495 and 4,552 triangular elements, respectively (right).

Capsules were assumed to be spherical with an outer radius of 2 mm (Figure 6). The spheres were divided into three compartments: a 0.17 mm thick membrane along the surface of the sphere, a liquid core and a cell pellet, each of the latter two occupying half of the core volume. To improve computational efficiency, the three-dimensional capsule was modelled in two dimensions by assuming rotational symmetry.

The capsule was meshed in Comsol Multiphysics 4.3 (Comsol AB, Stockholm, Sweden) using the built-in free triangular mesh algorithm, with extra fine element size in the membrane and the liquid core, and extremely fine element size in the cell pellet.

### Kinetic description of cell metabolism

Because the purpose of the mathematical modelling was only to visualise potential concentration gradients inside the capsules, yeast metabolism was modelled only with reactions consuming sugars and the inhibitors furfural and HMF. Thus, in the investigated time frames, growth and product formation were assumed to have negligible effects on the diffusion of sugars and inhibitors, and on the rates of consumption.

For the purpose of the model in this work, glucose uptake was modelled as a two-component system, consisting of a low and a high affinity transporter [25]. Xylose uptake was assumed to be facilitated by a single, low-affinity system [25]. Because they are transported by the same hexose transporter system, each sugar was assumed to competitively inhibit the uptake of the other sugar [26]. Xylose was assumed to inhibit both glucose uptake systems with the same inhibition constant [25]. Moreover, both uptake rates were assumed to be non-competitively inhibited by furfural, and to a lesser extent by HMF.

Consequently, glucose and xylose consumption rates (*v*_*g*_ and *v*_*x*_, respectively) were described by the rate equations

(1) $$\begin{array}{ll} v_{g} & =\left(\frac{v_{\mathrm{gmax},L}\;C_{g}}{K_{\mathrm{Mg},L}\left(1+\frac{C_{x}}{K_{\mathrm{ix}}}\right)+C_{g}}+\frac{v_{\mathrm{gmax},H}\;C_{g}}{K_{\mathrm{Mg},H}\left(1+\frac{C_{x}}{K_{\mathrm{ix}}}\right)+C_{g}}\right)\\ & \;\left(\frac{1}{1+\frac{C_{f}}{K_{\mathrm{if}}}}\right)\left(\frac{1}{1+\frac{C_{h}}{K_{\mathrm{ih}}}}\right) \end{array}$$

and

(2) $$v_{x}=\left(\frac{v_{\mathrm{xmax}}\;C_{x}}{K_{\mathrm{Mx}}\left(1+\frac{C_{g}}{K_{\mathrm{ig}}}\right)+C_{x}}\right)\left(\frac{1}{1+\frac{C_{f}}{K_{\mathrm{if}}}}\right)\left(\frac{1}{1+\frac{C_{h}}{K_{\mathrm{ih}}}}\right)$$

where *v*_*gmax*_ and *v*_*xmax*_ are the maximum specific rates of consumption (mmol/g dry cells/h); *C*_*g*_, *C*_*x*_, *C*_*f*_ and *C*_*h*_ are concentrations; *K*_*Mg*_ and *K*_*Mx*_ are half saturation constants; and *K*_*if*_ and *K*_*ih*_ are inhibition constants. Indexes *g*, *x*, *f* and *h* refer to glucose, xylose, furfural and HMF, respectively, while *L* and *H* refer to the low and high affinity glucose transport systems, respectively.

The specific rates of conversion of the furan aldehydes (*v*_*f*_ and *v*_*h*_) were described by Michaelis-Menten kinetics

(3) $$v_{f}=\frac{v_{\mathrm{fmax}}\;C_{f}}{K_{\mathrm{Mf}}+C_{f}}$$

(4) $$v_{h}=\frac{v_{\mathrm{hmax}}\;C_{h}}{K_{\mathrm{Mh}}+C_{h}}$$

### Boundary conditions and parameters

All bulk concentrations, as well as kinetic parameters and diffusivities used in the modelling, are summarised in Table 3.

**Table 3:** Bulk concentrations, kinetic parameters and diffusivities of glucose, xylose, furfural and HMF used as base case for mathematical modelling

	***C***_***bulk***_, **g/l**	***v***_***max***_, **mmol/g/h**	***K***_***M, ***_**mmol/l**	***K***_***i***_, **mmol/l**	***D***_***w***_, **m**^**2**^**/s**	***D***_***m***_, **m**^**2**^**/s**	***D***_***c***_, **m**^**2**^**/s**
Glucose	40	6 (L), 2 (H)	20 (L), 0.4 (H)	36 (xylose), 40 (furfural), 80 (HMF)	6.76 × 10^-10a^	6.08 × 10^-10^	3.38 × 10^-10^
Xylose	20	6	100	0.4 (glucose), 40 (furfural), 80 (HMF)	7.69 × 10^-10a^	6.92 × 10^-10^	3.85 × 10^-10^
Furfural	2	3	0.7		1.10 × 10^-9b^	9.90 × 10^-10^	5.50 × 10^-10^
HMF	2	1	20		1.06 × 10^-9c^	9.54 × 10^-10^	5.30 × 10^-10^

*Abbreviations*: *C*_*bulk*_ concentration in the bulk liquid; *D*_*c*_ effective diffusivity in the cell pellet; *D*_*m*_ effective diffusivity in the capsule membrane; *D*_*w*_ diffusivity in the bulk and in the liquid core of the capsule; *H* high affinity glucose transporter; HMF 5-hydroxymethylfurfural; *K* half saturation constant (Michaelis-Menten constant); *K*_*i*_ inhibition constant for each of the indicated compound; *L* low-affinity glucose transporter; *v*_*max*_ maximum specific consumption rate;

^b^Data from [27].

^c^Data from [28].

^d^Data from [29].

The biomass concentration inside the cell pellet compartment in the capsule was assumed to be constant at 300 g/l, as reported previously [15,30]. The concentrations of solutes in the bulk liquid outside the capsule were set as constant boundary conditions. Parameters for glucose and xylose transport were adapted from Lee *et al*. [25]. The inhibition constant for competitive inhibition of xylose uptake by glucose was assumed to be equal to the high affinity *K*_*M*_ value for glucose. For furfural consumption, the maximum rate and saturation constant identified during steady state conditions at high cell density in a membrane bioreactor were used [31]. HMF is generally consumed at a lower rate than furfural. It was assumed that the maximum rate of HMF conversion was one-third of the maximum furfural conversion rate, with a saturation constant of 20 mM [32-34].

Inhibition by furfural and HMF can mostly be observed as an extended lag phase in batch cultures, during which primarily furfural is converted to furfuryl alcohol. This indicates very strong inhibition, but cannot be modelled in the relatively simple kinetic framework of the present study. Sárvári Horváth and co-workers estimated the specific growth rate during pulse additions of furfural to an anaerobic continuous culture of *S. cerevisiae* CBS8066 [24]. By fitting the model

(5) $$\hat{\mu }=D\left(\frac{1}{1+\frac{C_{f,\mathrm{pulse}}/2}{K_{\mathrm{if}}}}\right)$$

to the measured specific growth rate using non-linear regression, we estimated the inhibition constant *K*_*if*_ is approximately 40 mM. In this equation, $\hat{\mu }$ is the estimated specific growth rate, *D* is the dilution rate equalling the specific growth rate at steady state prior to each pulse addition, and *C*_*f, pulse*_ is the concentration of the furfural immediately after pulse addition. In the absence of reliable data, and as HMF is known to be less inhibitory than furfural, we set the inhibition constant *K*_*ih*_ at 2 *K*_*if*_.

Diffusivities in the liquid bulk were taken from literature data (Table 3). The effective diffusivities in the liquid core, gel membrane and cell pellet were assumed to be 100%, 90% and 25% of the diffusivity in the liquid bulk (*D*_*w*_), respectively. The Sherwood number was set to 100 to exclude external mass transfer resistance.

### Efficiency factors and sensitivity analysis

To investigate the effect of encapsulation, the total rate of reaction in the two-dimensional cell pellet, *R* (mol/m/s), was calculated by surface integration for each of the four molecular species. An efficiency factor for each species *j*, *β*_*j*_, was calculated by dividing the resulting overall reaction rate, *R*_*j*_(*D*_*w*,*i*_), by the corresponding value obtained after increasing all diffusivities by a factor of 10^5^, *R*_*j*_(*D*_*w*,*i*_ × 10^5^), to emulate well-mixed conditions:

(6) $$\beta_{j}=\frac{R_{j\;}\left(D_{w,i}\right)}{R_{j}\left(D_{w,i}∙10^{5}\right)}$$

To assess the sensitivity of the system to one or a group of parameters, the total rates and efficiency factors were calculated at 10% lower and 10% higher values of the selected parameters. In analogy to metabolic response coefficients, the relative change in rates and efficiency due to the relative change in the parameter value, were calculated by

(7) $$C_{P}^{R_{j}}=\frac{d\;\ln R_{j}}{\mathrm{dlnP}}\approx \frac{\ln R_{j}\left(1.1P\right)-\ln R_{j}\left(0.9P\right)}{\;\ln1.1P-\ln0.9P}$$

(8) $$C_{P}^{\beta_{j}}=\frac{d\;\ln\beta_{j}}{\mathrm{dlnP}}\approx \frac{\ln\beta_{j}\left(1.1P\right)-\ln\beta_{j}\left(0.9P\right)}{\;\ln1.1P-\ln0.9P}$$

where $\;C_{P}^{R_{j}}\;$ and $C_{P}^{\beta_{j}}$ are the rate and efficiency response coefficients to the parameter *P*.

### Yeast strain

A xylose-fermenting strain, which we named CEN.PK XXX, was engineered in two steps using targeted homologous recombination of linearised DNA fragments directly into the yeast chromosomes XVI and VIII of *S. cerevisiae* CEN.PK 122 MDS [35]. In the first step, the copy number of enzymes in the pentose phosphate pathway was increased by integration of an additional copy of the *RPE1*, *TAL1* and *RKI1* genes. The promoter of the *TKL1* gene was also replaced with the glycolytic promoter of *ENO1*. The genetic construct was integrated into the N-terminal coding region of the *TKL1* gene in chromosome XVI to replace the native *TKL1* promoter. The *TKL1* coding sequence was left intact, and the *RPE1*, *TAL1* and *RKI1* genes were integrated upstream of the *TKL1* gene. The integration event was selected for by using the resistance gene *SMR1* (a mutant *ILV2*) and plating the transformed cells onto minimal medium agar plates with 50 μg/ml sulfometuron-methyl (Supelco, Bellefonte, PA, USA). In the second transformation, genes active in xylose metabolism were integrated: *XYL1* and *XYL2* from *S. stipitis*, and *XKS1* from *S. cerevisiae*. The synthetic codon-optimised coding segments of *XYL1*, *XYL2* and *XKS1* were each fused upstream with new promoter segments from −500 to −1 of three glycolytic enzymes: the promoter of *PGK1* with *XYL1*, the promoter of *TDH3* with *XYL2* and the promoter of *TPI1* with *XKS1*. The DNA segment containing the ‘xylose genes’ was flanked by segments of *GRE3*. The cells that had been transformed with the DNA fragment were transferred to a shake flask containing 20 g/l xylose in 2 × CBS medium [36], and after 5 days, clear growth was seen. The strain was maintained on minimal medium agar plates with 20 g/l D-xylose (Fisher Scientific, Leicestershire, England) as the only carbon source, and was used in all experiments.

### Media

The growth medium used for the batch cultivations was a defined growth medium (DGM), as previously reported [37], with ergosterol and Tween 80 (both Sigma, Steinheim, Germany) added during anaerobic cultivations, and glucose, xylose or a combination of the two as carbon source.

The hydrolysate used was made from spruce chips at 18 bar pressure for 5 to 7 minutes at pH 2.0 by addition of SO_2_, and kept refrigerated until use. Immediately prior to use, the pH was set to 5.5 with 10 M NaOH. The medium was autoclaved and centrifuged to remove solid particles. The final concentrations in the medium used for anaerobic fermentations were: glucose 12.8 ± 0.2 g/l, mannose 16.6 ± 0.3 g/l, galactose 3.6 ± 0.1 g/l, xylose 7.6 ± 0.1 g/l, arabinose 2.4 ± 0.0 g/l, acetic acid 3.1 ± 0.0 g/l and furfural 0.28 ± 0.08 g/l, after addition of salts, vitamins and trace metals as in the defined media.

### Encapsulation procedure

The capsules were prepared by the one-step, liquid droplet-forming method as described in [15]. In short, cells were grown in 100 ml DGM (40 g/l glucose and 20 g/l xylose) for 24 hours. Yeast was harvested from 50 ml of medium at OD_600_ of approximately 4 by centrifugation at 3500 × g for 4 minutes, and resuspended in 50 ml of 1.3% w/v sterile CaCl_2_ (Scharlau, Sentmenat, Spain) solution containing 1.3% w/v carboxymethylcellulose (Aldrich, Steinheim, Germany) with an average molecular weight of 250 kDa and degree of substitution of 0.9. Capsules were formed by dripping this solution through syringe needles into a stirred sterile solution of 0.6% w/v sodium alginate (catalogue number 71238; Sigma) and 0.1% v/v Tween 20 (Sigma-Aldrich, Steinheim, Germany). After gelling for 10 minutes, the capsules were washed with sterile ultrapure water, and hardened in 1.3% CaCl_2_. The capsules were subsequently submerged for 24 hours in 0.2% w/v low molecular weight chitosan (catalogue number 448869; Aldrich) solution with 300 mM CaCl_2_ in 0.040 M acetate buffer, pH 4.5.

### Cultivation and cell sampling

Propagation of encapsulated cells in capsules of approximately 15 ml was performed aerobically in 250 ml cotton-plugged conical flasks filled with 100 ml DGM containing 40 g/l glucose and 20 g/l xylose, which were incubated for 36 hours in a shaker bath (125 rpm) at 30°C. The capsules were rinsed with sterile 0.9% NaCl (Scharlau), and transferred to 100 ml DGM containing 5 g/l glucose and 40 g/l xylose, then incubated for another 24 hours prior to the start of the anaerobic batch fermentations. We chose these concentrations of glucose and xylose to ensure rapid cell growth by inclusion of a high concentration of glucose in the first step, and to maintain efficient xylose metabolism by using a low glucose and a high xylose concentration in the second step.

Propagation of suspended yeast was started with aerobic cultivation for 24 hours in 100 ml DGM (40 g/l glucose and 20 g/l xylose). Cells were harvested (3500 × g, 4 minutes) and resuspended to an OD_600_ of 3 in fresh medium of the same composition. After 36 hours, cells were again harvested and resuspended in 100 ml fresh DGM (5 g/l glucose and 40 g/l xylose) to an OD_600_ of 7.2, and cultured for another 24 hours of aerobic propagation. The residual sugar concentrations at the end of each propagation culture were similar. Hence, the propagation steps for the encapsulated and the suspended cells were as similar as practically possible.

Anaerobic batch cultivations were performed in conical flasks, equipped with a rubber stopper fitted with stainless steel capillaries for sample removal and a loop trap filled with sterile water to permit the produced CO_2_ to leave the flasks. Anaerobic encapsulated cultures were started by transferring 30 capsules to 120 ml of media containing combinations of glucose (0 or 40 g/l), xylose (0 or 40 g/l) and furfural (0, 1 or 2 g/l) (Sigma-Aldrich). This gave an initial cell concentration of 0.272 ± 0.014 g dry cells/l of liquid volume, meaning an average of 1.1 mg dry cells per capsule in the form of a visible pellet inside each capsule. Anaerobic cultivations of suspended cells were started at initial cell concentrations of 0.258 ± 0.030 g dry cells/l.

### Analytical methods, statistics, yields and elemental balance calculations

Metabolite concentrations were quantified by HPLC using an Aminex HPX-87H column (Bio-Rad, Hercules, CA, USA) at 60°C with 5 mM H_2_SO_4_ as eluent at a flow rate of 0.6 ml/min. A refractive index detector was used for the detection and quantification of glucose, xylose, xylitol, acetic acid, glycerol and ethanol. For the hydrolysate medium an Aminex HPX-87P (Bio-Rad), operated at 85°C with 0.6 ml/min ultrapure water as eluent, was used to quantify glucose, xylose, arabinose, galactose and mannose with a refractive index detector.

The cell dry weight was measured in predried and preweighed glass tubes. Cells were separated by centrifugation, and washed once with ultrapure water before drying for approximately 24 hours at 105°C. Cells from capsules were released by crushing the capsule, followed by extensive washing of the capsule debris with ultrapure water.

Yields of metabolites and biomass, as well as the carbon balance, were calculated from the determined concentrations at the end of the fermentations. The biomass composition, CH_1.76_O_0.56_ N_0.17_[38], was used in the carbon balance calculations. Error intervals are given as ± 1 standard deviation with n = 3, unless otherwise indicated.

## Abbreviations

DGM: Defined growth medium; FEM: Finite element modelling; HMF: 5-hydroxymethylfurfural; HPLC: high performance liquid chromatography; OD: Optical density;

### Symbols

*β*: Efficiency factor (−); *c*: Concentration (mM); $C_{j}^{R}$: Rate response coefficient to entity *j* (−); $C_{j}^{\beta }$: Efficiency response coefficient to entity *j* (−); *D*: Diffusivity (m^2^/s); *D*: Dilution rate (per h); *K*_*i*_: Inhibition constant (mM); *K*_*M*_: Half saturation constant (mM); n: Number of repeated experiment; *R*: Total rate of reaction in cell pellet calculated by surface integration (mol/m/s); *v*: Specific rate (mM/g/h); Y_Si_: Yield of compound *i* on consumed sugar (g/g);

### Subscripts

Ace: Acetate; *c*: Cell pellet; EtOH: Ethanol; *f*: Furfural; *g*: Glucose; Gly: Glycerol; *h*: 5-hydroxymethylfurfural; *H*: High affinity; *L*: Low affinity; *m*: Capsule membrane; *max*: Maximum; *P*: Parameter; *W*: Water; *X*: Xylose.

## Competing interests

The authors declare that they have no competing interests.

## Authors’ contributions

JOW contributed to the idea and experimental design of the study and the interpretation of results, carried out all fermentation experiments, and was the main author of the manuscript. NB designed and constructed CEN.PK XXX, and contributed to the writing of the manuscript. MJT contributed to the idea of the study and writing of the manuscript. CJF contributed to the idea and design of the study, created the final kinetic models and performed the simulations, and contributed to the interpretation of results and writing of the manuscript. All authors read and approved the final version of the manuscript.
